# A pilot study to determine the effect of one physical therapy session on physical activity levels for individuals with chronic low back pain

**DOI:** 10.1186/s13104-017-3006-x

**Published:** 2017-12-06

**Authors:** Wayne Brewer, Brian T. Swanson, Toni S. Roddey, Habeeblai Adewale, Caleb Ashmore, Jennifer Frerich, Cory Perrin, Alexis Ortiz

**Affiliations:** 1 Texas Woman’s University, 6124 Institute of Health Sciences-Houston, 7600 Fannin Street, Houston, TX 77030 USA; 2 University of New England, Portland, ME USA; 3 Texas Woman’s University, Houston, TX USA; 4 Harris Health Systems, Houston, TX USA

**Keywords:** Chronic low back pain, Physical activity, Physical therapy

## Abstract

**Background:**

A pilot study was conducted to quantify the effect size of changes in physical activity after of one session of physical therapy for individuals with chronic low back pain and to determine factors that predict daily sedentary activity time.

**Methods:**

Fourteen subjects with at least 3 days of physical activity accelerometer data were analyzed before and after one session of physical therapy. Data was analyzed using 1-tailed, paired t-tests with level of significance set at 0.05. Effect sizes were computed using the baseline and post intervention mean differences divided by the baseline and post-intervention differences in the standard deviation.

**Results:**

A nonsignificant reduction in steps-per-day and time spent performing sedentary activities, with increases in light and moderate–vigorous physical activity were found (effect size: 0.15–0.33). A nonsignificant decrease in daily sitting and standing time 1 week immediately following the physical therapy session and an increase in daily lying time (*p* = 0.03) (effect size: 0.23–0.69) were found.

**Conclusion:**

One physical therapy session resulted in a small physical activity change for individuals with chronic low back pain. Baseline and post intervention levels of pain catastrophisation and perceptions of disability need to be explored in future studies to determine if these are factors that influence levels of physical activity change for these individuals Results are limited by the small sample size, however the ability to increase physical activity in this population may be of clinical relevance.

*Trial Registration* NCT02823756; June 30, 2016: Retrospectively Registered

**Electronic supplementary material:**

The online version of this article (10.1186/s13104-017-3006-x) contains supplementary material, which is available to authorized users.

## Background

Low back pain (LBP) is a musculoskeletal problem that will affect approximately 80% of the population at some point of their lives [1] with an estimated unadjusted point prevalence ranging from 6.3 to 56.0 percent [2]. The impairments associated with LBP may progress to disability if they continue into a chronic state [3, 4]. Chronic low back pain (CLBP) is often due to repetitive overuse disorders but can also occur as the result of a one-time traumatic injury such as a fall or accident [5]. The continual disability suffered by individuals with CLBP is multifactorial. Vlayen and Linton noted that the fear avoidance model may partially explain why CLBP results in persistent disability for these individuals, due in part to increased pain catastrophizing and fear of movement [6]. Typically fear leads to hypervigilant behaviors to protect the individual from engaging in physical activities that are perceived as threatening [6, 7]. The result is the avoidance of necessary physical movements, normally used to perform instrumental activities of daily living. It was hypothesized that this lack of daily movement may lead to a continual cycle of elevated fear, catastrophizing, perceptions of disability, physical disuse and pain [6, 7].

Physical therapy (PT) is an integral component for the functional recovery of individuals with CLBP. Restoration of muscular strength, flexibility, spinal mobility and cardiovascular endurance are typically included in PT regimens designed to improve the function of individuals with CLBP [8]. Often these treatment programs are based on movement classification systems that attempt to categorized patients into distinct treatment paradigms such as: centralization/directional preference exercise, stabilization exercise, traction or manipulation based on factors including: the chronicity of the injury, presence of peripheral neurological symptoms, pain location, and provocation factors [8, 9]. In practice, there are many instances where the patients’ classification is unclear and has a non-specific pathoanatomical etiology that is hallmarked by recurrence of symptoms that often is debilitating [8]. Non-specific low back pain may be addressed with more than one treatment paradigm. A previous study by [10] found that only 50% of patients fit the described categories, with 25% fitting more than one category, and 25% not fitting any of the defined treatment classifications. This classification may be particularly difficult in individuals with longer duration of LBP [11]. Despite the lack of evidence for a standardized exercise prescription for individuals with CLBP, the use of progressive graded exercise has been shown to increase physical activity [12, 13]. No published studies have examined if physical activity patterns are altered after one session of exercise training for individuals with CLBP.

Spinal manipulation has been shown to have mild to moderate short-term improvements on perceptions of pain and disability that can occur after one treatment session [14]. The proposed rationales for these improvements include a wide range of effects, such as neurophysiological changes, increased segmental joint mobility, and placebo effects from the procedure [15]. Despite these reported benefits, spinal manipulation, which has been shown to be highly effective for individuals with acute low back pain, appears to be less effective for individuals with subacute and chronic low back pain [8, 14, 16]. However, previous studies that have assessed the effectiveness of spinal manipulations used subjective reports of pain and self-perceived levels of disability [14, 16]. The impact of spinal manipulation on objective measures of physical activity is not clear when performed on a CLBP population.

There are a multitude of patient misconceptions regarding the effectiveness of diagnostic and treatment modalities that are utilized in the medical model such as advanced imaging, opiate use, analgesic/anti-inflammatory injections and surgical procedures [17–20]. Strong, documented evidence for any of these diagnostic and treatment interventions is lacking and frequently these procedures are palliative in nature [19, 20]. Accordingly, education is considered to be of paramount importance for individuals with CLBP [21]. Patient education delivered in the context of PT interventions typically focuses on three key areas: (1) addressing the fear avoidant behaviors displayed by the patient; (2) informing the patient regarding basic pain science principles; and (3) applying cognitive behavioral approaches such as graded activity and graded exposure programs to promote confrontation with the perceived threat to the patient’s well-being [6, 13, 22]. These educational methods are often combined with biomechanical principles to promote safe activity performance to prevent re-exacerbation of symptoms. Patient education that utilize cognitive behavioral approaches are often combined with other interventions and are dispersed over several treatment sessions that use patient self-perceptions of pain and disability as the outcomes studied [21, 23–26]. To date, there are no studies that examined if there are immediate changes in physical activity patterns for individuals who receive an initial session of PT that is comprised of patient education.

Despite the myriad of rehabilitative and medical interventions used to address the pain, impairments and resultant disabilities for individuals with CLBP, the documented effectiveness for any one treatment paradigm is lacking [17, 27]. CLBP is often viewed as recalcitrant to interventions [1, 8, 20, 28]. Most published studies utilize self-perceptions of pain and disability as the primary endpoint [29]. Others use the aforementioned measures combined with physical performance measures such as walking tests, handgrip strength, muscular strength and spinal mobility assessments [30]. While there are published studies that examine the levels of physical activities (PA) for these patients using reliable methods for PA monitoring such as accelerometry, the majority of these studies have occurred outside the clinical environment [31–38]. Accelerometry uses small devices worn on the hip or wrist that measure movement, change of position, steps per day and energy expenditure for a given time period [39–42]. The accuracy of accelerometers far exceeds self-reported questionnaires of PA which often suffer threats to validity such as recall bias [43–45].

When assessed via accelerometer, studies suggest that there are no differences in levels of physical activity between individuals who have chronic pain when compared to healthy, age-matched controls [35, 38]. It is difficult to determine from these studies if the subjects had increased levels of fear avoidance, pain catastrophisation or self-perceptions of disability, however most of these studies were cross-sectional in design. There is a dearth of literature that examines short term changes in objectively measured PA when physical therapy interventions, particularly spinal manipulations, exercise and patient education are administered to these individuals. Studies are needed to quantify if there is an effect of these commonly used physical therapy interventions on free-living physical activity for individuals with CLBP. Free-living physical activity is defined as “the level of activity that the patients, within their physical limitations, at their own pace, and in their own environment, typically perform [46]”.

A pilot study was conducted to examine the effects of physical therapy interventions for individuals with CLBP based on the aim to quantify the short-term effects of one PT session that included spinal manipulations, exercise and patient education on free-living PA in individuals with CLBP. This combination of interventions represents a “typical” initial physical therapy session of individuals with CLBP. We hypothesize that the additive effects of each intervention will have the capacity to have an immediate increase in free-living physical activity. The purpose of this pilot study was to determine effect sizes that may be used to establish sample sizes for future studies that investigate the efficacy of physical therapy interventions to increase physical activity in persons with CLBP.

## Methods

Subjects were recruited from a publicly funded, hospital-based outpatient physical therapy clinic. The inclusion criteria were: (1) patient referral to outpatient PT with a CLBP related diagnosis; (2) CLBP without radiating pain distally to the knee > 3 months in duration; (3) ability to read and write in English or Spanish; and (4) between the ages of 18 and 70 years old; (5) able to ambulate independently without assistive devices. Subjects were excluded if they had: (1) previously been diagnosed via radiography or clinical exam with spinal instability, fracture or tumor; (2) a clinical indication of nerve root pathology; (3) previous spinal surgery; (4) a diagnosis of osteoporosis or rheumatoid arthritis; (5) used oral steroids within the previous 6 months; (6) a workman’s compensation or disability claim filed for a previous low back injury; (7) self-report of current or suspected pregnancy; and (8) presented with incomplete accelerometer data. All subjects completed an informed consent document prior to enrollment into the study that was approved by the Institutional Review Board of Texas Woman’s University and Harris Health Systems.

### Outcome measures

*Physical activity* Triaxial accelerometers [GT3XP-BTLE; Actigraph, LLC., FL, USA] were used to measure the physical activity level of the subjects at a frequency of 30 Hz. This is a small device with dimensions that are 4.6 cm × 3.3 cm × 1.5 cm, that weighs 19 g. The inclinometer within these accelerometers was also activated to measure time spent in sitting, standing or recumbent postures. The accelerometers were activated within the Actilife software [v6.0; Actigraph, FL, USA] using each subject’s weight, height, race/ethnicity, sex, date of birth, and hand dominance. The means of the following parameters were the variables of interest for this study: (1) number of steps taken each day; (2) mean percentage of the day spent performing sedentary (SED) [(0–99 counts), light (LHT) (100–1951 counts), moderate-to-vigorous physical activity (MVPA) (≥ 1952 counts) each day; 3] mean percentage of the day spent in the standing, lying and sitting positions. The Actigraph accelerometer has excellent reliability and validity with other methods for assessing energy expenditures across varied levels of physical activity [47]. The subjects were instructed to wear the accelerometer on the right hip during their waking hours for at least 8 h for a period of 7 days. Instances where the accelerometer did not reach values higher than zero counts within a 10-min epoch were considered as non-wear time. If the data for each subject did not reach the pre-established wear time of at least 3-days, then the data for this subject was removed from the analysis. Therefore, in order to consider data valid for analysis, each subject needed to have at least 5 h/day of wear time for at least 3 days during the 7-day period, regardless of whether the days were consecutive or not. Three days of accelerometry data has been suggested in other studies to be the minimal wear time to reliably capture physical activity patterns in adults [48–50]. The percentage of time spent performing SED, LHT and MVPA per day were calculated with the Freedson 1998 algorithms [51].

### Study protocol

Six physical therapists participated in this study. Their clinical experience ranged from 2 to 8 years. All of them received advanced training in orthopedic manual physical therapy with a patient caseload comprised of approximately 90–95% orthopedic disorders with approximately 50% of those patients referred to physical therapy with low back pain. Patients were referred to the outpatient physical therapy clinic to be evaluated for their primary complaint of low back pain. During this process, eligibility for inclusion into the study was assessed by the physical therapist assigned to the patient. If the patient was found eligible for inclusion in the study, he or she was invited to participate in the study; all patients who accepted the invitation then completed the informed consent process. The first session included data collection only, with no treatment intervention conducted during this session. To obtain baseline data, each subject was asked to wear the accelerometer for the next seven consecutive days for at least 8 h/day. Each subject was scheduled for their first treatment session one week after the initial evaluation to allow for one full week of PA data capture utilizing the accelerometer. At the first treatment session, the accelerometer was retrieved and the data downloaded onto a designated research computer via ActiLife^®^ software. To ensure adequate retrieval of the accelerometer, subjects who missed the first treatment session were either called or emailed to reschedule the appointment. Subjects who returned for the first treatment session without the accelerometer were asked to bring it to the next treatment session; the data was analyzed using only the initial 7 days from the day of issue. If a subject did not: (1) return the accelerometer; (2) return it with adequate data; (3) return for a scheduled physical therapy session; and (4) respond to the text, email or phone messages, then the patient was considered lost to follow-up.

The physical therapy intervention was based on a previously published clinical guideline on the management of low back pain [8]. The physical therapy intervention consisted of one treatment session that included a manipulation technique(s) to either the sacroiliac joint, thoracic, or lumbar spine. The manual therapy interventions are described below and pictures and descriptions of the manual therapy interventions can be found in Additional file 1: Appendix S1.

1. Thoracic gapping manipulation: a high-velocity, low amplitude end-range technique was delivered using an anterior–posterior directed thrust at the mid and lower thoracic spine using the patient’s crossed arms and flexed elbows.
2. Lumbopelvic gapping manipulation: a high-velocity, low-amplitude end-range thrust technique was delivered using an anterior-inferior directed thrust applied to the flexed lumbar spine in a side-lying position.
3. Lumbopelvic unilateral gapping mobilizations: a mid to end-range, non-thrust mobilization technique applied to lumbar spine in side-lying using an anterior-lateral directed force with the individual’s cranial hip flexed.
4. Hip long-axis distraction manipulation: a high-velocity, end-range thrust technique applied in an axial direction through the distal lower extremity to the flexed, abducted and slightly externally-rotated hip joint in supine.

Each subject was instructed on an exercise program based on the clinical judgement of the physical therapist that are classified as motor control exercises, transversus abdominis training, lumbar multifidus training, and dynamic lumbar stabilization exercises based on the published clinical guidelines by [8]. The primary exercises included were: (1) quadruped heel rocks, supine abdominal brace and bent knee fallout exercises to enhance motor control, recruitment of the transversus abdominis and multifidi; (2) chair stands, seated hip hinge, bridging were used to promote dynamic lumbar stabilization during functional movements. The pictures and descriptions of the exercises can be found in Additional file 1: Appendix S2. The sequence of how the manual therapy and exercise interventions were administered can be found in Additional file 1: Appendix S3.

These exercises were performed at the first treatment session and each patient was instructed in a home exercise program to promote increased segmental mobility and stability of the lumbar spine. Each subject performed a submaximal aerobic endurance exercise on either a bicycle, treadmill or elliptical trainer with the duration and intensity set at a moderate intensity level based on the effort that was verbally given to the therapist. Patient education was provided which consisted of techniques to promote self-management of his or her CLBP condition via cognitive behavioral approaches such as graded exercise, graded exposure or basic pain science information to minimize the hypervigilant behaviors such as restriction of certain activities [12, 13, 21]. At the conclusion of the first treatment session, each participant was issued the accelerometer for a second time and instructed to wear the device in a similar fashion for another 7 day period. Each subject was asked to return the accelerometer at their next treatment session, scheduled 7 days later.

### Data analysis

The means and standard deviations (SD) for the following physical activity variables derived from the accelerometer were computed: number of steps taken each day, percentage of the day spent performing SED, LHT and MVPA per day (counts/day) and the percentage of the day spent in the sitting, lying and standing positions. Descriptive variables such as the mean age, height, weight, BMI and gender of the study participants were calculated. Cohen’s *d* was calculated to determine the baseline and post intervention effect size for the variables previously listed using the formula below:

$$d\,\, = \,\,\frac{{{\text{Mean}}_{{({\text{baseline}})}} \,\,{-}\,\,{\text{ Mean}}_{{({\text{post}}\, - \,{\text{intervention}})}} }}{{{\text{SD}}_{{({\text{pooled}})}} }}$$

The aim of this pilot study was to determine the effect size of one physical therapy session on physical activity levels, one-tailed paired t-tests were performed with the level of significance set at 0.05 determine if significant differences were found between the baseline and post-intervention means for the aforementioned variables. One-tailed level of significance supports the hypothesis that the one session intervention will promote a decrease in the SED activities while promoting an increase in LHT and MVPA. Twenty-seven subjects were needed to achieve 80% power based on an a priori analysis using an effect size of 0.50 for 0.05 level of significance using one-tail. The effect size used for the power analysis was based on a systematic review published by Keller et al. that examined the effects of interventions performed on individuals with chronic low back pain using self-perceptions of pain and function as the outcomes [52]. They reported pooled effect sizes of 0.57, 0.52 and 0.35 for behavioral interventions, exercise training and manipulation, respectively.

## Results

**Fig. 1:**
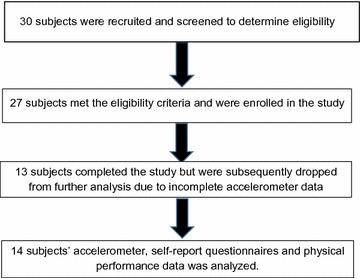
Consort diagram of flow of subjects in the study

Thirty subjects were screened for eligibility, with 27 subjects fully enrolled. After screening for validity of the accelerometer data, a total of 14 subjects’ accelerometer, self-report and physical performance measures were analyzed (Fig. 1).

The 13 subjects that were not included in the analysis did not meet the required wear time of the accelerometer as previously discussed. There were 11 females and 3 males (7 African-Americans, 6 Hispanic, 1 Caucasian); the mean age and BMI was 50.2 years, 31.6 kg/m^2^, respectively. The physical activity measures and wear time of the accelerometer at baseline and post intervention are presented in Table 1.

**Table 1:** Mean (S.D.) percentage of the day spent performing: sedentary, light, moderate–vigorous physical activities and standing, lying and sitting position

	Baseline, (n = 14)	Post-intervention	Effect size (*d*)	*p*-value
Steps per day	5735.1 (3484.9)	5205.7 (3374.3)	0.15	0.22
SED (%)	62.1 (9.5)	59.0 (10.9)	0.31	0.12
LHT (%)	35.7 (8.2)	38.5 (9.1)	0.33	0.13
MVPA (%)	2.1 (2.2)	2.5 (2.8)	0.16	0.28
Standing (%)	40.4 (11.4)	37.9 (9.9)	0.23	0.07
Lying (%)	9.46 (6.7)	16.1 (11.9)	0.69	0.03
Sitting (%)	50.1 (10.8)	46.0 (13.0)	0.34	0.11
Wear time (min)	732.0 (123.1)	698.8 (142.6)		0.395^¥^

^¥^Denotes 2-tail analysis

There was a trend towards reduction in the number of steps taken per day and the time spent performing SED activities, with concurrent increases in LHT and MVPA. These findings presented with small effect sizes that ranged from 0.33 and.16 for the percentage increase in time spent performing LHT and MVPA, respectively. The paired t-tests revealed no post-intervention significant differences from baseline in physical activity levels (Table 1). A nonsignificant decrease in percentage of the day was spent sitting and standing during the 1 week immediately following the physical therapy session, with a concurrent significant increase in the percentage of the day spent lying (*p* = 0.03). The baseline to post-intervention effect sizes for the percentage of time spent in each position were small to medium and ranged from 0.23 to 0.69 (Table 1).

## Discussion

The purpose of this pilot study was to determine the effect size that one session of PT has on physical activity. This pilot study showed that one session of PT that included spinal manipulation, exercise and patient education had a trend towards increasing physical activity for individuals with chronic low back pain. There was a small effect of reducing the percentage of time spent performing SED activities with a similar effect of increasing time spent performing light physical activities. Paradoxically, the percentage of time spent lying increased while the time spent standing and sitting decreased.

Researchers that conduct studies that examine the impact that PT has on free-living physical activities need to carefully consider their aims of their study to be adequately powered. If the aim of their study is to determine the impact that PT has on reducing time spent performing SED and increasing LHT physical activities for individuals with CLBP then the sample size requirements are approximately four times less than studies that seek adequate power to detect changes in MVPA. Despite MVPA being touted as the level of activity needed to promote health, studies that examine changes in this type of behavior must have the resources needed to support a large number of subjects to detect small changes in this behavior. It is evident that this drastic difference in sample size requirements stems from the fact that for individuals with CLBP, the majority of the time is spent performing SED activities which provides researchers a larger opportunity to study the effects of interventions to shift these individuals’ physical activity levels towards the light intensity.

There are several limitations of the study that warrant discussion. The extremely small sample size limits the ability to make inferences to a larger population. This study was underpowered due to non-compliance with accelerometer wear time. Despite the increased risk of bias, the power analysis was based on previous effect sizes determined for pain and self-perceptions of function, which are variables not considered for this study. Because this is the first study to examine changes in physical activity levels after one physical therapy session, there were no established effect sizes that could be used to directly determine the sample size to be sufficiently powered. The subjects in this study were primarily female, whose ethnic backgrounds were predominately African-American or Hispanic, and who were seeking physical therapy services at a publicly-funded outpatient clinic. Previous studies suggest that leisure-time physical activity patterns tend to be lower for: men compared to women, minorities compared to Caucasians, and individuals with lower as compared to those of higher socioeconomic status [53, 54]. The results in this population may not be consistent with those observed in other populations. Lastly, causation would have been more evident with the addition of a control group using a mixed between and within subjects design, however the present design did allow for each subject to be his or her own control. Future studies that examine the impact of physical therapy on physical activities patterns need to utilize larger, more ethnically diverse sample sizes with individuals of varied socioeconomic levels with longer follow-up periods.

## Conclusion

There have been numerous studies that have examined the short-term effects of physical therapy interventions on the understanding of pain neurophysiology, self-reported pain, disability and biomechanics. To our knowledge, this is the first study to determine the effect size of a single physical therapy session that includes exercise, patient education and spinal manipulation on changes in physical activity profiles for individuals with CLBP. The generation of these effects sizes will allow future researchers to determine adequate sample sizes needed to answer additional research questions regarding the impact of physical therapy on physical activity. A comprehensive physical therapy program results in a small effect to increase the level of physical activity in individuals with CLBP as soon as the first treatment session.

## Additional files

**Additional file 1: Appendix S1.** Lumbopelvic manipulation techniques.

**Additional file 2: Appendix S2.** Lumbar stabilization and range of motion exercise program.

**Additional file 3: Appendix S3.** Physical therapy treatment sequence.

## Abbreviations

LBP: low back pain
CLB: chronic low back pain
PT: physical therapy
PA: physical activity
SED: sedentary
LHT: light
MVPA: moderate–vigorous physical activity
