# Supplementary material for: A pilot study to determine the effect of one physical therapy session on physical activity levels for individuals with chronic low back pain
Source: BMC Res Notes. 2017 Dec 6;10:691. doi: 10.1186/s13104-017-3006-x (PMC5717841; doi:10.1186/s13104-017-3006-x)

Appendix S1

**Thoracic gapping manipulation**: patient is positioned in supine. A high-velocity, low amplitude end-range techniques with an anterior-posterior directed thrust at the mid and lower thoracic spine using the patient’s crossed arms and flexed elbows. The therapist uses an open palm or closed fist to stabilize the selected segment and uses the sternal region to push down on the patient’s flexed elbows in order to manipulate the selected segment.


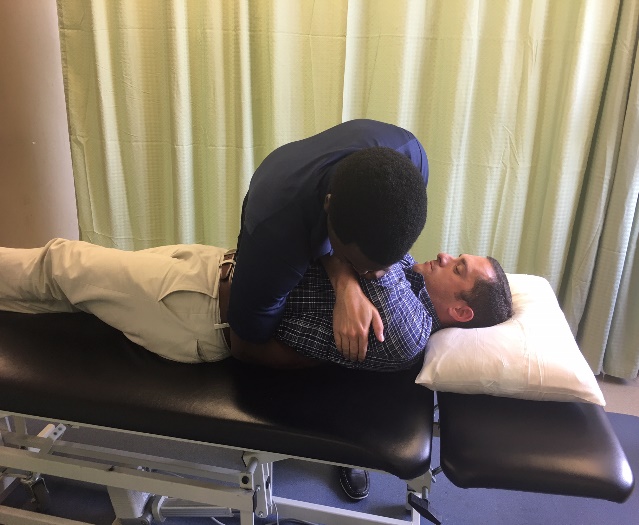


**Lumbopelvic gapping manipulation:** a high-velocity, low-amplitude end-range thrust technique in an anterior-inferior direction applied to the flexed lumbar spine in a side-lying position. The lumbar segment is localized, followed by a thoracic flexion/rotation to stabilize cranial segments. An end range thrust technique is performed with the therapist’s forearms.


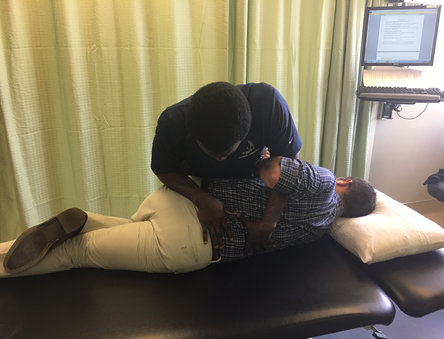


**Lumbopelvic unilateral gapping mobilization:** a mid to end-range, non-thrust mobilization technique applied to lumbar spine in side-lying in an anterior-lateral direction with the individual’s cranial hip flexed similar to the lumbopelvic gapping technique described previously. The lumbar segment is localized, followed by a thoracic flexion/rotation to stabilize cranial segments. A mid to end-range mobilization is performed.

**Hip long-axis distraction manipulation**: a high-velocity, end-range thrust technique applied through the distal lower extremity to the flexed, abducted and slightly externally-rotated hip joint in supine. The lower extremity is placed in 30 degrees or hip flexion, hip abduction, and 5degrees of external rotation and a longitudinal distraction technique is applied thru the lower extremity.


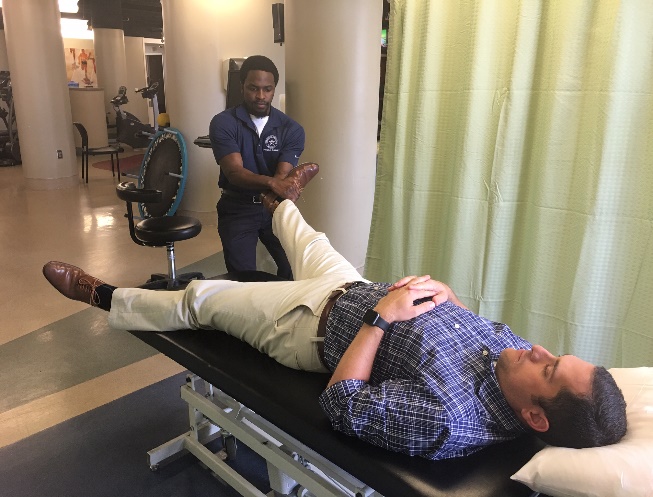

Supplement: Supplementary file 1 — Additional file 1: Appendix S1. Lumbopelvic manipulation techniques. [file 13104_2017_3006_MOESM1_ESM.docx]
