# Supplementary material for: A pilot study to determine the effect of one physical therapy session on physical activity levels for individuals with chronic low back pain
Source: BMC Res Notes. 2017 Dec 6;10:691. doi: 10.1186/s13104-017-3006-x (PMC5717841; doi:10.1186/s13104-017-3006-x)

Appendix S2

**Quadruped heel rocks**:

The patient assumes a quadruped positon and keeps his/her ankles in a plantar-flexed position. While maintaining a flat lumbar spine, the patient proceeds to sit back on the heels and maintains this position for a few seconds.


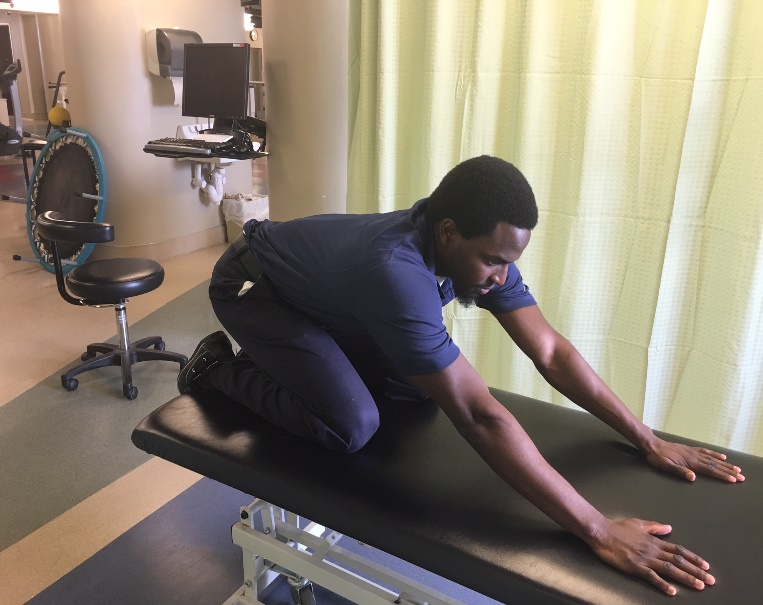


**Seated hip hinge**

Patient assumes an erect sitting posture and then proceeds to hinge/bend forward at the hips while maintaining the lumbar spine in a neutral position. The patient proceeds to increase hip flexion until he/she is unable maintain the spine as a unit. The patient returns to the starting position and repeats.


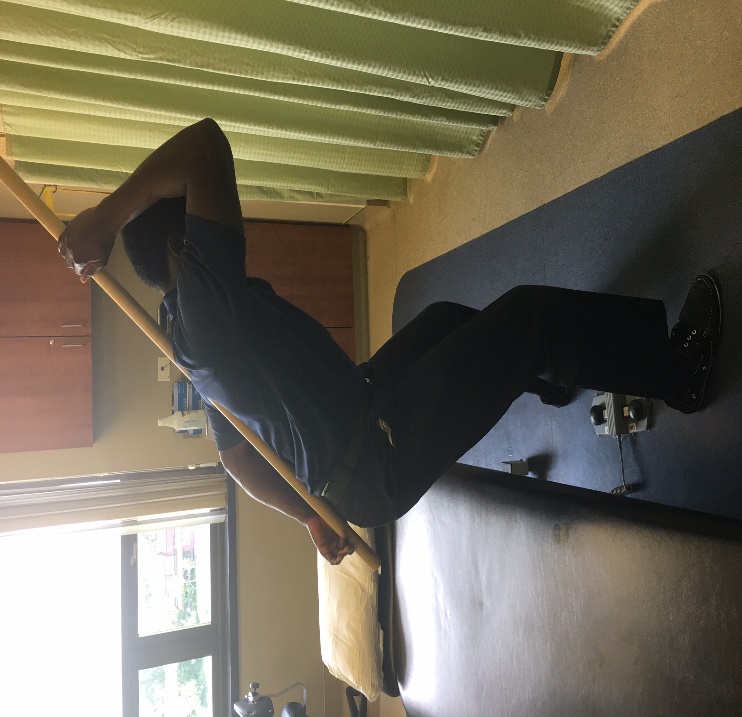


**Supine bridge**

While in a supine position, the patient bends both knees to approximately 90 degrees. The patient then proceeds to contract the abdominal and gluteal muscles. The patient lifts the buttock of the ground as to create a “bridge” with the body.


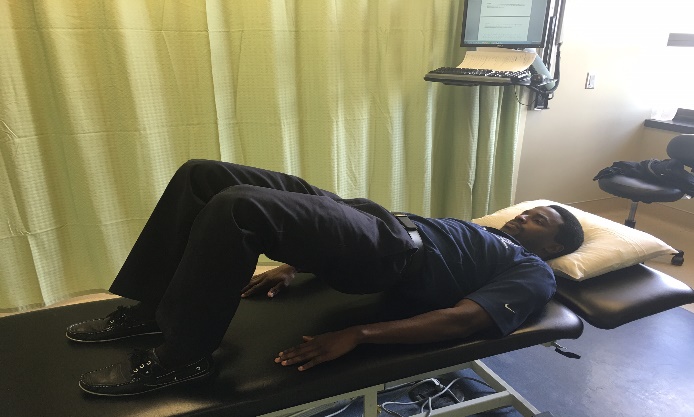


**Chair stands**

The patient starts by sitting in a chair or treatment table with an erect posture. The patient then flexes both shoulders to 90 degrees and proceeds to lean forward by employing a hip hinge as described previously as the abdominal and lower extremity muscles contract as the patient assumes the standing position without the use of the upper extremities.

**Supine abdominal brace**

Patient lies supine with hips and knees flexed to 90 degrees with a blood pressure cuff placed underneath the lumbar spine and inflated to 40 mmHg. The patient draws their abdomen upwards and inwards, raising the cuff pressure to 50 mmHg and holds for 5 seconds and then repeats.

**Supine bent knee fall out**

Patient assumes the same position and draws the abdomen upwards and inwards as described above followed be alternating hip abduction/lateral rotation from flexion. The therapist monitors the lumbar spine and pelvis to ensure there it does not rotate within the first 50% of lower extremity motion while maintaining the cuff pressure between 50-60 mmHg.


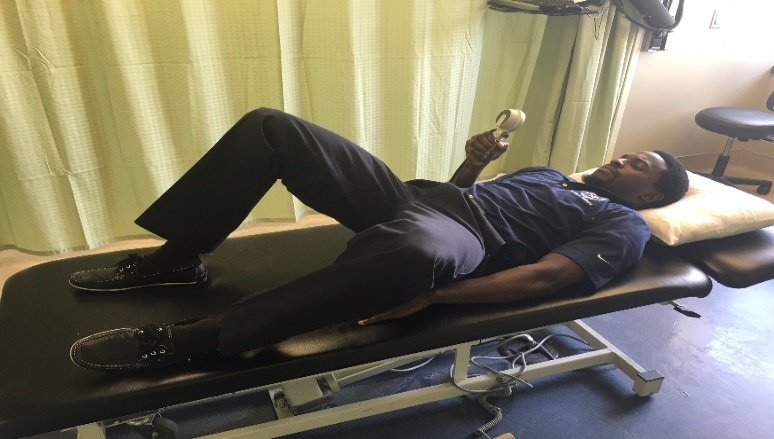

Supplement: Supplementary file 2 — Additional file 2: Appendix S2. Lumbar stabilization and range of motion exercise program. [file 13104_2017_3006_MOESM2_ESM.docx]
