# Supplementary material for: A pilot study to determine the effect of one physical therapy session on physical activity levels for individuals with chronic low back pain
Source: BMC Res Notes. 2017 Dec 6;10:691. doi: 10.1186/s13104-017-3006-x (PMC5717841; doi:10.1186/s13104-017-3006-x)
Supplement: Supplementary file 3 — Additional file 3: Appendix S3. Physical therapy treatment sequence. [file 13104_2017_3006_MOESM3_ESM.docx]

Appendix S3

1. The treatment encounter commenced with a determination of the specific movement(s) of that reproduces the patient’s familiar pain.
2. If it was determined that the patient’s lumbar mobility was limited as a specific segment, or lumbar range of motion was limited in specific direction(s), or if pain was reproduced with a specific movement, a targeted lumbar manipulation was performed as described in Appendix I.
3. The specific movement(s) that reproduced the patient’s pain was then reassessed to determine if a change in symptoms has been met.
4. A moderate intensity aerobic activity using cardiovascular exercise equipment such as the treadmill, elliptical trainer or bicycle was performed at a rating of perceived exertion that did not exceed a 3 on a 1 to 10 scale for 10-12 minutes was performed prior to commencing the exercise program.
5. The primary exercise program as described in Appendix II were commenced.
